# Optical coherence tomography as a marker of neurodegeneration in patients with Wilson’s disease

**DOI:** 10.1007/s13760-017-0788-5

**Published:** 2017-05-09

**Authors:** Ewa Langwińska-Wośko, Tomasz Litwin, Karolina Dzieżyc, Michał Karlinski, Anna Członkowska

**Affiliations:** 1 Department of Ophthalmology, Medical University of Warsaw, Warsaw, Poland; 2 2nd Department of Neurology, Institute of Psychiatry and Neurology, Sobieskiego 9, 02-957 Warsaw, Poland

**Keywords:** Wilson’s disease, Optical coherence tomography, Neurodegeneration, Unified Wilson’s Disease Rating Scale

## Abstract

Wilson’s disease (WD) is an inherited autosomal recessive disorder that leads to pathological copper accumulation in different organs. Optical coherence tomography (OCT) is proposed as a marker of neurodegeneration in many neurological diseases. Thinning of the total retinal nerve fiber layer (RNFL) and macular thickness (Mth) examined by OCT was detected in patients with WD, especially those with brain magnetic resonance imaging changes. The aim of this study was to evaluate the relationship between OCT parameters and the progression of neurological signs measured by the Unified Wilson’s Disease Rating Scale (UWDRS) in patients with WD. Consecutive patients with WD admitted to the Department of Neurology underwent OCT to assess the thickness of the macula and total RNFL. Patients also had neurologic assessments according to the UWDRS part III. Patients were divided into two groups based on the presence (UWDRS+) and absence (UWDRS−) of neurological symptoms. Fifty-eight patients (34 females, 24 males) were enrolled. Mean duration of treatment was 9 years (standard deviation [SD], ±10.8). The mean UWDRS score at the time of study was 8.4 (range 1–52; SD ±13.9) points. Total RNFL as well as macula thickness were significantly decreased in the UWDRS+ group versus the UWDRS− group. A significant negative correlation was found between OCT parameters (RNFL and Mth measurements) and neurological impairment according the UWDRS scale. This study confirms that OCT may be a useful tool for measuring the degree of neurodegeneration in patients with WD, and may play role in monitoring disease progression.

## Introduction

Wilson’s disease (WD) is an inherited autosomal recessive disorder that leads to copper accumulation in the liver, brain, cornea, and other organs. The most common ophthalmological sign is the Kayser–Fleischer ring as a result of accumulation of copper in the cornea [1, 2]. Sunflower cataract is a rare ophthalmological manifestation of patients with WD that is sometimes observed during the course of the disease [3].

Recent studies detected morphological and functional disturbances in visual pathways in patients with WD using optical coherence tomography (OCT), electroretinography, and visually evoked potentials [4, 5]. OCT is a non-invasive imaging technique that provides objective assessments of the retinal nerve fiber layer (RNFL), which is mainly composed of unmyelinated axons [6, 7]. Measuring the thickness of the RNFL with OCT provides an objective estimate of the integrity of these axons. Thinning of the RNFL as a result of axonal loss was previously proposed to be a marker of neurodegeneration in many diseases including WD [8–10].

A previous study conducted in patients with WD also found retinal thinning in these patients when examined using OCT [4, 8]. Brain magnetic resonance imaging (MRI) changes in patients with WD reflects nervous system injury [11–15]. A recent study showed that thinning of the macula and RFNL was especially pronounced in the group of patients with WD who had MRI changes [4].

Our hypothesis assumed that damage to the retina and RNFL also reflects the degree of neurological impairment in patients with WD. Thus, the aim of this study was to evaluate the relationship between OCT parameters and the degree of neurological impairment assessed by the Unified Wilson’s Disease Rating Scale (UWDRS) in patients with WD [16].

## Materials and methods

### Patients

This study was a cross-sectional, non-interventional, observational study approved by the Medical University of Warsaw Bioethics Committee. All participants provided informed consent. The manuscript follows the Advised Protocol for OCT Study Terminology and Elements recommendations for reporting quantitative optical coherence tomography studies [17].

The study population consisted of consecutive WD cases admitted during a 2-year period to the Second Department of Neurology of the Institute of Psychiatry and Neurology, Warsaw Poland. All patients had a confirmed diagnosis of WD according to established international criteria [1].

Ocular exclusion criteria were (i) contraindication to the use of 1% tropicamide, (ii) previous ocular surgery, (iii) a history of ocular trauma, and (iv) significant pathology of the anterior and posterior eye segments, refractive error of more than ±1.5 diopters (3). To rule out the above-mentioned conditions, all patients underwent a routine ophthalmological assessment by a single experienced ophthalmologist, including best corrected acuity for distance and near, intraocular pressure, examination of anterior segment of the eye (slit lamp), and pupil dilated fundus. One patient was excluded from the study because of refractive error of −1.75 D sph with myopic retina degeneration. In addition, in single patient, one eye was not considered eligible due to a history of cataract extraction followed by retinal detachment.

All included participants underwent detailed neurological examination according to the UWDRS and OCT to assess the thickness of the macula and the total RNFL (described below). Finally, patients were divided into two groups according to the presence (UWDRS+) or absence (UWDRS−) of neurological symptoms as assessed by the UWDRS.

### Neurological assessments

The neurological evaluation was based on a detailed neurological examination. Patients were scored using the UWDRS to assess the severity of neurological signs and symptoms. For the analysis, part III of the UWDRS was used (0 points for no neurological involvement and 143 points for maximum severity of neurological deficits) [16].

### Optical coherence tomography

OCT was performed in one place (Department of Ophthalmology, Medical University of Warsaw, Poland), on the same day as the qualifying ophthalmological examination by experience operator using a Spectralis OCT (Cirrus HD-OT Spectral Domain Technology, Zeiss, Germany) with software version 5.11. Under moderate light conditions and after dilatation of pupils, both eyes of each patient were examined. Total thicknesses of the macula (Mth) and RNFL were measured separately.

The protocol for macula thickness generates a macular cube 512 × 128 of data through a 6 mm square grid by acquiring a series of 128 horizontal scan lines each composed of 512 A-scans, except for the central high definition vertical and horizontal scans, which was composed of 1024 A-scans each. Macular images were manually segmented to determine the thickness of specific layers of the retina at the macula: the ganglion cell and inner plexiform layer complex (GCIP), the inner nuclear layer (INL), the outer plexiform layer (OPL), and the outer nuclear layer plus the photoreceptor layer (ONL + PRL). The thickness of all layers was measured at the thickest point within perifovea, with the exception of the ONL + PRL, which was measured at the center of the fovea. To maximize accuracy, manual segmentation was performed on the black-and-white images.

The RNFL measurement was performed after manually centering the optic disc. The protocol RNFL thickness generates a cube of data through a 6 mm square grid by acquiring a series of 200 horizontal scan lines each composed of 200 A-scans. Thickness values for four quadrants (superior, temporal, inferior, and nasal) were automatically generated by the software.

### Statistical analysis

To avoid internal correlations, data were analyzed by patients using mean values of measurements taken from both eyes, except for a single patient in whom only one eye was eligible. The data are presented as means and standard deviation (SD). The normality of the distribution of each variable in each group was assessed using the Shapiro–Wilk test. Comparisons of the OCT and UWDRS were performed using Student’s *t* test (if variables fulfilled the assumptions of normal distribution) or Mann–Whitney test (if variables did not fulfill the assumptions of normal distribution). Univariate correlations between each of the OCT variables and UWDRS were evaluated using Spearman’s correlation coefficients. For patients with neurological signs and symptoms (UWDRS+), a regression model was performed to assess the relationship between results in UWDRS part III and OCT parameters.

Statistical analysis was performed using the computing environment R (R Development Core Team 2005). *p* values below 0.05 were considered statistically significant.

## Results

Fifty-eight patients (34 females and 24 males) were enrolled in the study. The mean age at diagnosis was 29.5 (SD ±11.3) years, mean age at the study start was 38.5 (SD ±12.5) years, and the mean duration of treatment was 9 (SD ±10.8) years. Thirty patients were treated with d-penicillamine and the remaining 26 with zinc sulfate.

The UWDRS+ group consisted of 30 patients. The remaining 28 were without neurological features at the time of the examination (UWDRS−). The mean UWDRS score at the time of the study was 8.4 (range 1–52; SD ±13.9) points.

The results of the OCT examination differed between the UWDRS+ and UWDRS− groups. Patients from the UWDRS+ group had an RNFL layer that was thinner by 6.71 µm (*p* = 0.002).

Thickness values for the four quadrants concerned the temporal quadrant (T) and were different between the UWDRS+ and UWDRS− groups by 7.91 um. This quadrant mainly reflects the results of the RNFL total value (Table 1).

**Table 1:** Results of ocular coherent tomography (OCT) in patients with Wilson’s disease with neurological symptoms Unified Wilson’s Disease Rating Scale (UWDRS+) and those without neurological feature (UWDRS−)

Parameter	UWDRS+ (*N* = 30)		UWDRS− (*N* = 28**)**		*p* value	Difference
	Mean	SD	Mean	SD		
RNFL (µm)	88.05	8.39	94.76	7.43	0.002	−6.71
Retinal nerve fiber layer measurements						
S	110.00	10.84	115.13	13.01	0.082	−
T	61.74	9.38	69.65	10.42	0.005	−7.91
I	115.20	12.07	120.81	11.16	0.082	–
N	70.41	8.97	74.17	10.43	0.162	–
Macular thickness measurements						
Mth (µm)	254.46	16.39	268.35	19.76	0.007	−13.89
GCIP	78.59	3.26	81.93	3.59	0.001	−3.34
INL	32.81	2.28	35.89	2.51	>0.000	−3.08
OPL	30.70	1.30	30.85	1.43	0.693	–
ONL + PRL	118.91	3.84	11,989	4.73	0.406	–

*GCIP* ganglion cell and inner plexiform layer, *I* inferior part, *INL* inner nuclear layer, *Mth* total macular thickness, *N* nasal part, *ONL* + *PRL* outer nuclear layer and photoreceptor layer, *OPL* outer plexiform layer, *RNFL* retinal nerve fiber layer, *S* superior part, *SD* standard deviation, *T* temporal part

The total Mth in the UWDRS+ group was significantly decreased in comparison with the UWDRS− group by 13.98 µm (*p* = 0.007).

Some segmented layers of the retina in the macula region were also thinner. The GCIP and the INL were different by 3.34 µm (*p* = 0.001) and 3.08 µm (*p* < 0.001), respectively (Table 1). No differences in the outer pOPL or ONL + PRL were found.

**Fig. 1:**
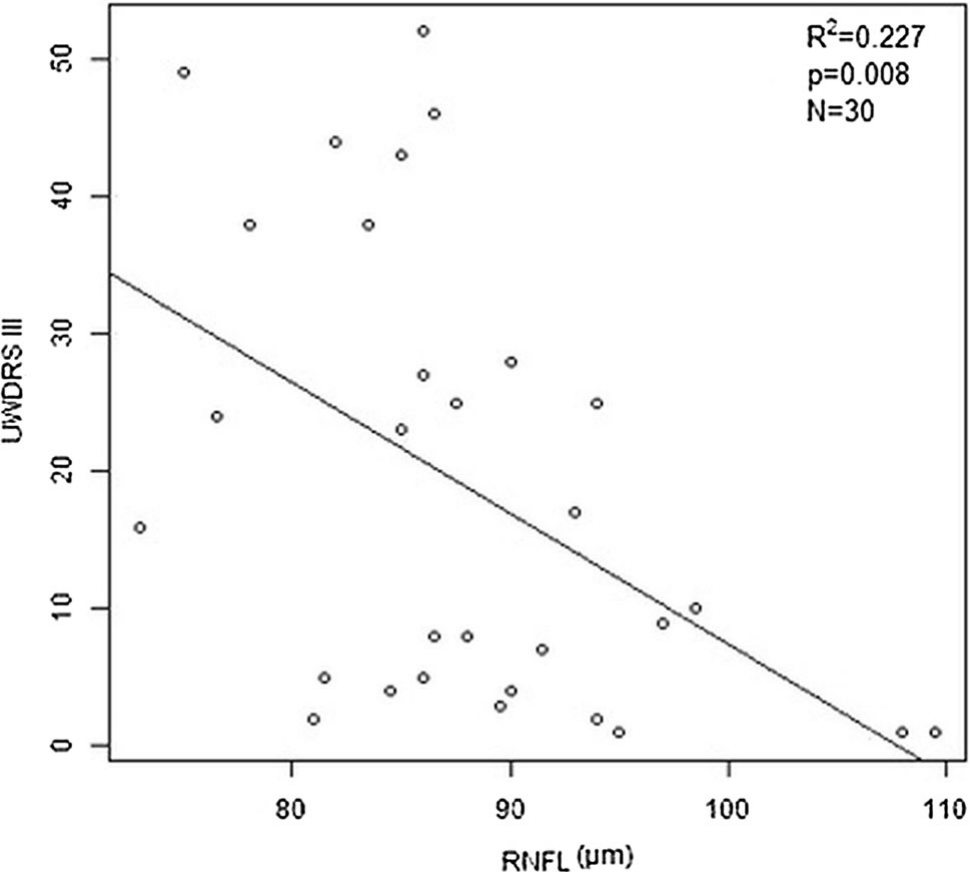
Linear relationship between the Unified Wilson’s Disease Rating Scale (UWDRS) part III score and the thickness of the total retinal nerve fiber layer for the UWDRS+ group

A significant negative correlation was found between the UWDRS part III score and the thickness of the total RFNL in the regression model (−0.951; *p* = 0.008) (Fig. 1).

**Fig. 2:**
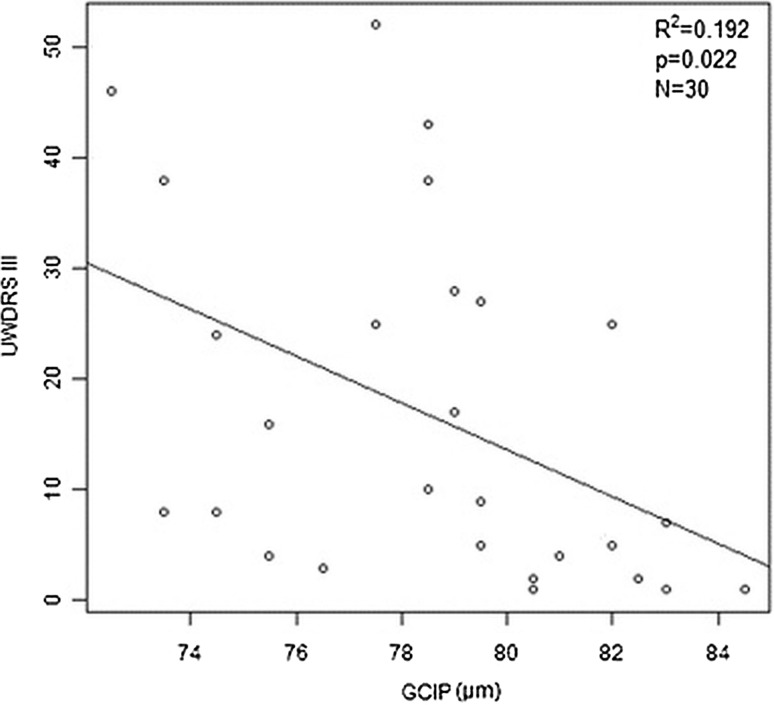
Linear relationship between the Unified Wilson’s Disease Rating Scale (UWDRS) part III score and the thickness of the retina in the ganglion cell and inner plexiform (GCIP) layer in Wilson’s disease patients (UWDRS+)

Similarly, a significant negative correlation was found between the scores on the UWDRS part III and some Mth layer measurements in the GCIP and OPL (−2.112 [*p* = 0.022] and −4.803 [*p* = 0.04], respectively) (Fig. 2).

## Discussion

Our study shows that there is a negative correlation between structural damage in the retina and the UWDRS scale score. This proves the relationship between degeneration of the retina, which is an element of the central nervous system, and other impairment of the nervous system affected by WD. The results show the possible role of OCT as a potential non-invasive technique that can be used together with brain MRI [4] and UWDRS to assess recovery, progression, and monitor treatment in patients with WD.

Of note, in patients with WD who have neurological symptoms, OCT parameters were significantly worse than in those without neurologic features. The morphological changes in the retina influence its proper function. A previous study conducted on patients with WD showed that this degeneration causes retardation of conductivity along the visual pathway, which was confirmed by prolonged VEP latency [4].

Deposits of copper accumulate over the course of the disease and cause injuries to different organs [1, 2, 18]. Pathological changes in the brain in patients with WD are mainly located in the basal ganglia, but copper may accumulate in other central nervous system structures [11, 13]. The retina consists of axons and glia without myelin, and may be good structure for visualizing the degree of neurodegeneration [19]. Albrecht et al. first analyzed the retinal structure in patients with WD and the mean thickness of the peripapillary RNFL, which was thinner and especially pronounced in the lower quadrants. According to the authors, RNFL thinning, as well as the thickness of the retinal ganglion cell and the internal plexiform layer complex, are suggestive of abnormalities within the ganglion cells and their axons [8].

OCT as a marker of disease progression is widely used in patients with multiple sclerosis (MS) [4]. The parameters of OCT were proven to correspond with the clinical subtype of MS. RNFL thinning was more pronounced in patients with a progressive MS disease course [20, 21]. Reduced RNFL thickness also was more advanced in patients with brain atrophy [22, 23].

OCT as potential marker of disease progression also was investigated in patients with Huntington’s disease (HD). Similar to our study, OCT results in patients with HD showed that the temporal RNFL is preferentially affected. The authors concluded that this is possibly caused by mitochondrial dysfunction. In addition, temporal RNFL thickness occurrence was inversely correlated with disease duration. Mth was significantly inversely correlated with motor impairment and disease duration [9].

Concerning retinal thickness and Parkinson’s disease (PD), RFNL thinning correlated with the duration of disease and its severity [24, 25]. One study found a significant negative correlation between the RNFL thickness in the right nasal superior quadrant and the Unified Parkinson Disease Rating Scale (UPDRS) score [10].

## Conclusions

OCT may be a useful biomarker for assessing neurological symptom progression and for monitoring course of WD.
